# Highly Emitting Perovskite Nanocrystals with 2-Year Stability in Water through an Automated Polymer Encapsulation for Bioimaging

**DOI:** 10.1021/acsnano.2c01556

**Published:** 2022-08-01

**Authors:** Sahitya Kumar Avugadda, Andrea Castelli, Balaji Dhanabalan, Tamara Fernandez, Niccolo Silvestri, Cynthia Collantes, Dmitry Baranov, Muhammad Imran, Liberato Manna, Teresa Pellegrino, Milena P. Arciniegas

**Affiliations:** ^†^Nanomaterials for Biomedical Applications and ^‡^Nanochemistry, Istituto Italiano di Tecnologia, Via Morego 30, 16163 Genova, Italy; § Instituto Interuniversitario de Investigación de Reconocimiento Molecular y Desarrollo Tecnológico (IDM), Universitat Politècnica de València-Universitat de València, Camino de Vera s/n, E46022 València, Spain

**Keywords:** perovskite nanocrystals, polymer, water stability, automated fabrication, bioimaging

## Abstract

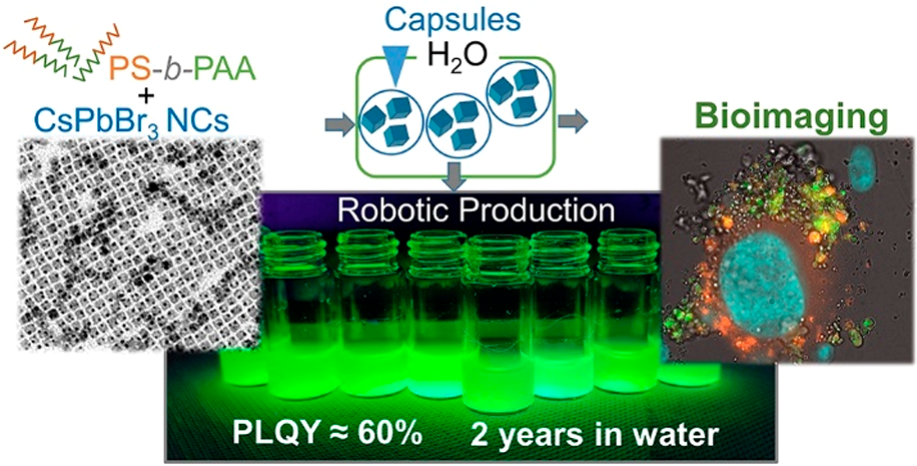

Lead-based halide perovskite nanocrystals are highly luminescent materials, but their sensitivity to humid environments and their biotoxicity are still important challenges to solve. Here, we develop a stepwise approach to encapsulate representative CsPbBr_3_ nanocrystals into water-soluble polymer capsules. We show that our protocol can be extended to nanocrystals coated with different ligands, enabling an outstanding high photoluminescence quantum yield of ∼60% that is preserved over two years in capsules dispersed in water. We demonstrate that this on-bench strategy can be implemented on an automated platform with slight modifications, granting access to a faster and more reproducible fabrication process. Also, we reveal that the capsules can be exploited as photoluminescent probes for cell imaging at a dose as low as 0.3 μg_Pb_/mL that is well below the toxicity threshold for Pb and Cs ions. Our approach contributes to expanding significantly the fields of applications of these luminescent materials including biology and biomedicine.

All inorganic metal-halide perovskite nanocrystals (NCs) with Cs^+^ as a site cation (for example, CsBX_3_; X: Cl^–^, Br^–^, or I^–^, and B: divalent metal cation) have been intensively investigated in photovoltaics^1^ and lighting^2,3^ owing to their tunable bandgap,^4^ large absorption coefficient,^5^ and close to unity photoluminescence quantum yield (PLQY).^6,7^ In addition, they feature high defect tolerance and color emission tunability by controlling the halide composition.^8^ Importantly, such structures can be prepared through simple synthetic protocols at relatively low temperatures.^9^ Their flexible processing has made it possible to prepare NCs with different sizes, shapes, and ligand coating.^10^ These characteristics make such NCs an ideal material system to achieve long-term high emission stability. Although such NCs feature better thermal stability compared to organic–inorganic structures, the obstacles for their progress toward commercialization reside in (i) the ionic character of the structure that makes them sensitive to water/moisture and (ii) the presence of lead as metal cation with its related toxicity issues.^11^ Indeed, the rapid degradation of perovskite NCs when exposed to water and moisture has strongly delayed their practical applicability. Therefore, many strategies have been reported to preserve the NCs for longer times at ambient conditions by isolating them from the environment. Examples are polymer-SiO_2_ shelling,^12^ amphiphilic polymer or solid–lipid encapsulation,^13,14^ synthesis of structures with reduced dimensionality,^15^ doping,^16^ use of bulky ligands,^17−22^ incorporation of NCs in a polymeric matrix,^23−27^ or their preparation within polymeric nanoreactors,^28,29^ which had recently allowed reversible halide exchange.^30^ While all these techniques have clear advantages, it remains a challenge to simultaneously preserve the high PLQY and synthetic flexibility of the original colloidal NCs while also providing a stable dispersion in water over long times. Moreover, current protocols require a long preparation time, multiple encapsulation steps in different materials, and their scalability has not yet been proved. These factors together also prevent the progress toward the use of these NCs in applications such as X-ray detection and low-dose imaging, biosensing, and bioimaging.^26,31,32^ In this context, perovskite NCs offer stronger photostability with significantly high PLQY (near 100%), narrower and more symmetric full-width at half-maximum (fwhm) than traditional organic dyes, relatively long PL lifetime, and importantly, they can be fabricated through simple and low cost synthesis protocols, which entails a number of reduced steps compared to other systems such as metal chalcogenides NCs and fluorescent proteins.^10,33^ Changes of synthesis conditions and post-treatments can also be easily performed to prepare crystals with different colors of emission and to extend their emission up to the near-infrared.^34^ Moreover, as shown by in vivo studies in plant cells, epidermal cells have a rapid and effective mechanism for detoxifying lead that involves the endoplasmic reticulum. This mechanism may account for the lower toxicity of Pb-based perovskites in comparison with traditional Cd-based quantum dots.^35^ Together, these material’s features benefit their potential application for multicolour bioimaging.^36^ However, their long-term stabilization in such conditions with a good compromise on optoelectronic properties still needs to be addressed (see Table S1 that summarizes the stability over time of different encapsulated systems that have been recently reported). Improving the stability of perovskite NCs in water could also boost their practical application in, for example, electrocatalysis, light emitting diodes (LEDs), lasing, and inks for printable electronic devices. In parallel, the development of automated procedures that enable the rapid fabrication of perovskites NCs in water is crucial for both screening purposes and for scaling up their production, as demonstrated in other systems,^37−40^ including the robust preparation of NCs/polymer nanobeads where small reaction volumes are needed.^41,42^ Compared to bench protocols, such routines are also more cost-effective since less time and less manual lab work are required.

In this work, we report the fabrication of CsPbBr_3_ NCs in-capsules that retain a PLQY of ∼60% for over two years in water through a few series of fabrication steps that are scalable by using an automated routine. As encapsulating amphiphilic polymer we chose polystyrene-*block*-poly(acrylic acid) (PS-*b*-PAA) at low molecular weight (34 kDa). This polymer in the presence of the NCs in toluene and upon addition of methanol (MeOH) in a single phase system enables the formation of polymer capsules containing the NCs and their transfer to different polar solvents. This protocol does not rely on prior surface modification of the as-synthesized NCs and is insensitive to their surface coating. Therefore, our protocol works for both NCs coated with cesium oleate (Cs-oleate) and NCs coated with didodecyldimethylammonium bromide (DDAB). However, the oleate molecules, with a reduced steric hindrance compared to DDAB ones, likely allow a better intercalation of PS, providing a tighter insulation of the NCs and thus a more reliable platform for our protocol in terms of PLQY values. Next, we demonstrate the robustness of the capsules, as their emission was found to be stable over 259 days in a saline solution (under physiological conditions compatible with biological materials). Furthermore, we assessed the cytotoxic effect of the Cs-oleate coated NCs in-capsules on an in vitro cell model and found that there is no significant toxicity in cell viability assay after 72 h of materials exposure (0.3 μg_Pb_/mL). Additionally, at this capsule dose we could clearly localize the luminescent capsules within the cancer cells by confocal imaging using such a low concentration of Pb in the capsules that is well below the Pb toxicity threshold.

## Results/Discussion

### Capsule Fabrication

**Figure 1:**
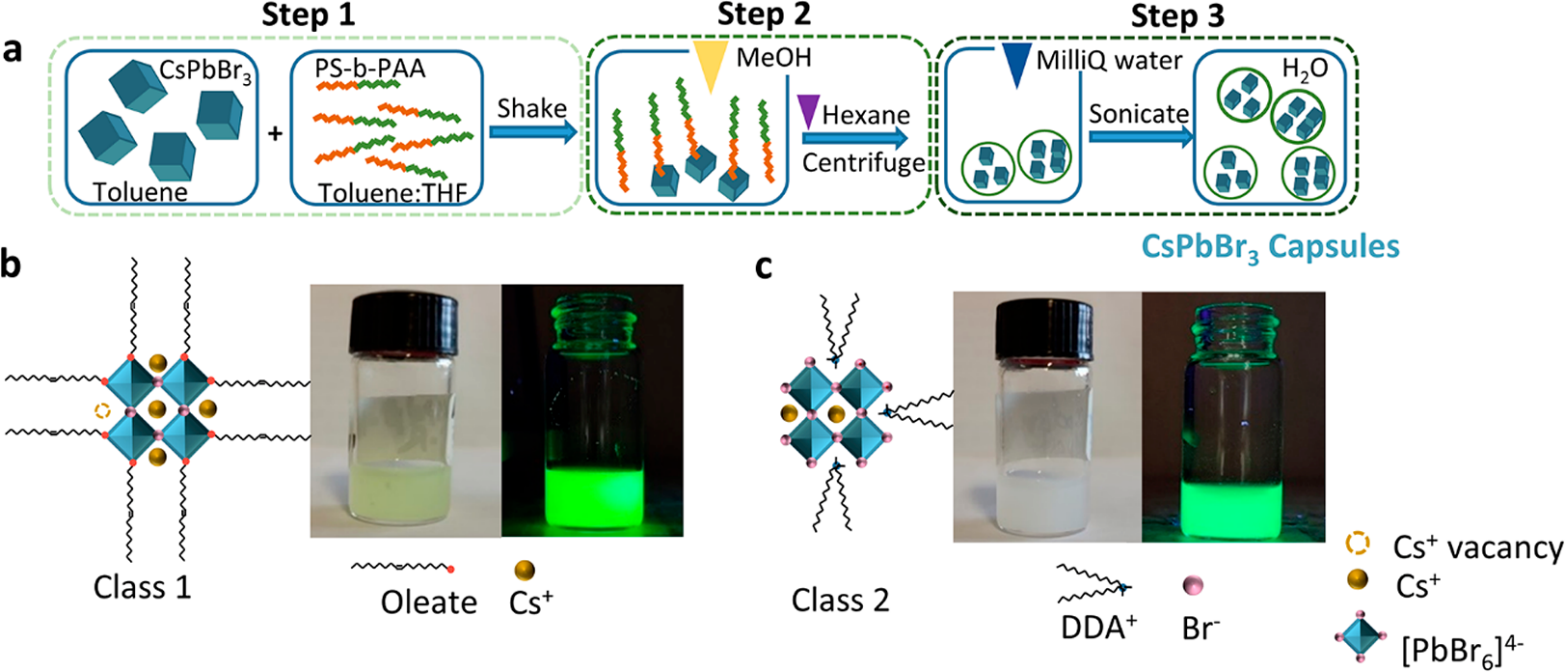
(a) Schematic illustration of the room temperature process developed for the fabrication of the capsules embedding the CsPbBr_3_ NCs. (b, c) Photographs of aged samples of (b) Cs-oleate and (c) DDAB-coated CsPbBr_3_ NCs in-capsules dispersed in Milli-Q water under normal and UV light evidencing that the samples remain highly emitting after long time of incubation in water. The sketches in (b) and (c) highlight the surface coating of the Cs-oleate (class 1) and DDAB (class 2) initial NCs used in the encapsulation experiments. The photographs in (b) and (c) were collected (b) 18 and (c) 24 months after their respective synthesis. Photographs of the same solutions soon after their preparation are displayed in Figure S14.

We selected two sets of CsPbBr_3_ efficient emitting nanocubes of ∼9 nm edge length that were synthesized in set 1 by using oleic acid and secondary amines and in set 2 by ligand exchange of the NCs from set 1 with DDAB, as reported previously by our group.^43,44^ The resulting NC’s surface from set 1 is coated with Cs-oleate (further referred to as class 1) and that from set 2 is coated with DDAB (further referred to as class 2). The initial samples are dispersed in toluene, and they have the typically reported cubic shape (Figure S1 of the Supporting Information, SI). The choice of these two sets of samples was based on their high PLQY.^43^ In contrast with DDAB-coated nanocubes, Cs-oleate-coated CsPbBr_3_ NCs tend to degrade quickly over time and thus their PLQY decreases to ∼10% after 21 days in toluene.^43^ Thus, we used freshly prepared samples in our experiments, and their concentration was determined based on the content of Pb via elemental analysis using inductively coupled plasma optical emission spectroscopy (ICP-OES, see Methods/Experimental). The Pb concentrations are reported in Table S2. For the encapsulation, we used PS-*b*-PAA with a lipophilic polystyrene (PS) polymer chain of 29 kDa and a poly(acrylic acid) (PAA) one of 5 kDa that was dissolved in a toluene/tetrahydrofuran (THF) mixture with a low content of THF (Methods/Experimental). After mixing the selected sample of NCs and PS-*b*-PAA polymer under shaking, the encapsulation was triggered by controlled addition of MeOH. Here, the polymer, initially in a nonpolar solvent (toluene), gradually rearranged upon addition of the more polar solvent (MeOH) at a well-defined flow rate, forming capsules where the PS block linked to the NC’s surface through interaction with their hydrophobic ligand shell, while the polar PAA block stretched outward, ensuring dispersibility in polar media. Next, the capsules were precipitated out of the toluene/THF/MeOH mixture by addition of hexane as antisolvent. After centrifugation, the capsules pellet was redispersed in water by vigorous sonication (see Methods/Experimental). The steps for the fabrication of the capsules are illustrated in Figure 1a, and the photographs of the capsules dispersed in water under a UV lamp are shown in Figure 1b and c. The capsule solutions appear as milky dispersions with bright green PL (when exposed to ultraviolet (UV) light) that is preserved for months (Figure 1b,c).

**Figure 2:**
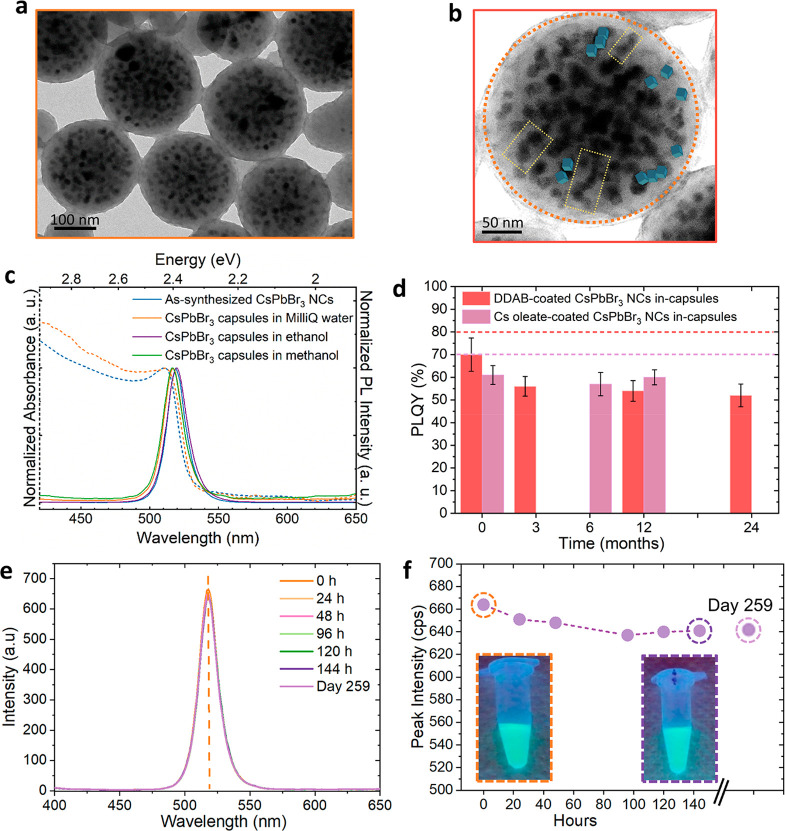
(a) TEM image showing a group of capsules containing the CsPbBr_3_ NCs at their core. (b) Magnified TEM view of a single capsule. The embedded sketches highlight the randomly dispersed NCs and the assembled aggregates. Examples of these aggregates are framed in yellow. (c) Representative normalized PL (solid) and absorbance (dashed) spectra collected from the as-synthesized class 2 NCs (DDAB-coated CsPbBr_3_ NCs) in toluene and the corresponding capsules dispersed in different polar solvents. (d) PLQY tracking of capsules dispersed in water over months. Values represent mean ± standard deviation (SD) of five independent measurements on the selected samples. The dotted lines in the figure indicate the PLQY of the respective initial NCs in toluene before encapsulation. (e) PL spectra and (f) emission intensity vs time of the Cs-oleate coated CsPbBr_3_ NCs in-capsules incubated in saline solution (0.9% NaCl) over a period of 259 days. The insets in (f) show the incubated capsules under UV light at day 0 (framed in orange) and after 144 h (framed in dark violet).

Figure 2a displays a representative transmission electron microscopy (TEM) image that shows the formation of well-defined capsules with a round shape that contain multiple aggregated NCs as well as single ones, which are located at the core of the capsule and wrapped by a polymer shell of ∼25 nm thickness (Figure 2b). Such NC aggregation is likely formed due to the NC’s incompatibility with MeOH and the partial incompatibility of the polymer with this solvent.^45,46^ We also observe that there is a fraction of capsules with a low or no NC content (mostly formed by polymer) (see Figure S2).

From the TEM images, the diameter of the capsules deposited from water is 250 ± 80 nm, while their average hydrodynamic size measured via dynamic light scattering (DLS) directly in water is 491 ± 80 nm (by intensity weight percentage; see Figure S3) with an average zeta potential value of −43 ± 3 mV. As expected, the larger capsule size detected via DLS is due to the hydration sphere formed around the polymer capsules by the highly swellable and negatively charged PAA polymer block.

The photoluminescence (PL) spectra collected from the samples excited at 350 nm confirm their emission with a peak centered at around 518 nm with a (fwhm) of ∼18 nm (Figure 2c), as reported for CsPbBr_3_ NCs coated with Cs-oleate or DDAB.^43^ The PL spectrum collected from the Cs-oleate coated CsPbBr_3_ in-capsules in Milli-Q water is displayed in Figure S4. The absorbance profile of the capsules dispersed in water agrees with that from the as-synthesized NCs (Figure 2c), confirming that the encapsulation does not alter their structure, as it is also evidenced by the collected X-ray diffraction patterns (Figure S5).

### Automated Capsule Fabrication

The scalability of colloidal NCs synthesis and their post synthetic processing is essential to reduce their fabrication cost and time, allowing a broader use of such materials. Therefore, we performed a partial automation of our benchtop polymer encapsulation protocol using a robotic platform to make the procedure less laborious and substitute manual addition of solvents with automated dispensing. A sketch illustrating the adopted steps of the protocol is displayed in Figure S6, and the detailed routine is described in the Supporting Information. To implement the scaling up, we choose a straightforward replication of the bench protocol in multiple vials using the Nimbus automated system (Figure S7) previously implemented for the synthesis of metal halide perovskite single crystals.^40^ The transfer of the fabrication to an automated liquid handling was facilitated by the steps developed on the on-bench protocol. As a result, we achieved a time-efficient and reproducible robotic encapsulation process that worked on various batches of Cs-oleate coated CsPbBr_3_ NCs in-capsules (see Figures S8 and S9). The automated procedure enabled us to produce 24 batches of capsules per run while keeping the temperature and rate of reagent mixing consistent across all the batches. Compared to the on-bench protocol through which 4–5 samples can be prepared within 5 h, the automated routine allowed the fabrication of 96 samples in less than 90 min (Figure S10). This approach is critical to produce large volumes of samples at high concentrations that are tedious to achieve through the on-bench protocol performed by a human. The PLQY values of the samples produced by our automated routine had an average value of 55 ± 6.0% (Table S3), comparable to the PLQY values obtained from the samples prepared manually. The collected PL spectra from this set of capsules are shown in Figure S11 and the measured DLS and Zeta-potential in Figure S12. Together, these results confirm that our encapsulation protocol can be made time-efficient by simply employing a robotic system without compromising the quality of final product, a routine that could guide the development of similar automated ones for other materials.

### Capsules Robustness in Different Solvents and over Long Time of Incubation in Water

The robustness of the fresh capsules was tested by redispersing them separately in three different solvents, water, ethanol, and MeOH, and by studying their PL over time. The capsules preserved their emission after 1 h of strong shaking in the corresponding solvents (Figures 2c and S4) and after 2 weeks in ethanol and MeOH (Figure S13 for Cs-oleate samples). Next, we focused on the stability of the capsules dispersed in water, as this is the most challenging solvent for preserving perovskite NCs. We measured the PLQY of the two classes of fresh samples before (in toluene) and after their encapsulation (in water). A detailed description of the protocol used for the evaluation of the PLQY is given in the Methods/Experimental section. Before encapsulation, we obtained 70% and 80% PLQY for class 1 and class 2 NCs, respectively. After encapsulation, the NCs retained over 85% of the initial PLQY, with 61% for Cs-oleate coated CsPbBr_3_ NCs in-capsules and 70% for DDAB-coated CsPbBr_3_ NCs in-capsules when following the same encapsulation protocol. During the optimization of our protocol, we found that the accurate selection of solvents and the proper sequence of their addition played a major role in preventing damage to the encapsulated NCs. Although THF is a good solvent for both PS and PAA blocks of the PS-*b*-PAA, the optical stability of the initial NCs in such solvent was limited to a few minutes. This significantly hampered the PLQY of the final capsules prepared through the same protocol when using THF for dissolving the polymer, which resulted in PLQY values of only 15% ± 10%. Therefore, to preserve the optical and structural stability of the NCs, we used a mixture of toluene, which is a very good solvent for the NCs, and THF (only at 10% in volume) to dissolve well the polymer. Note that at the polymer concentration used in the experiments, toluene alone did not easily dissolve the polymer as when using a low content of THF. Another aspect that proved crucial to obtain capsules with high emission stability was the gradual change of solvent from toluene to MeOH to water via precipitation with hexane. The controlled first addition of MeOH to the toluene/THF mixture of NCs and PS-*b*-PAA gradually increases the solution polarity, already promoting the formation of capsules, as evidenced by the change from a clear solution to a milky scattering dispersion. However, because of the presence of toluene (and THF), the PS block swells in solution, and the direct addition of Milli-Q water to the capsules (either as an addition to the current toluene/THF/MeOH mixture or as a solvent after capsules precipitation through centrifugation) quenched the NC’s emission. This was prevented by the introduction of an intermediate step in the process in which the capsules were precipitated via hexane addition and subsequently redispersed in pure MeOH. The use of MeOH as polar solvent (which has an affinity toward the PAA, but not toward the PS block) helps to complete the polymer packing around the NCs with the desired insulating conformation where the NCs/PS core is fully wrapped by a PAA shell. On the other hand, the surface functionalization of CsPbX_3_ NCs with quaternary ammonium ions such as DDAB is known to stabilize the perovskite NCs compared to NCs prepared with primary^47,48^ or secondary amines.^43,49^ Strikingly, in our process, we observed that the PLQY values measured from different batches of Cs-oleate NCs-in capsules had a narrower variance compared to that of DDAB ones (see data in Table S4), denoting a higher reproducibility of our process for the Cs-oleate samples from batch to batch. This can be explained by a better intercalation of the PS block given the less steric hindrance of Cs-oleate that provides a better surface stability and thus a narrower variation in the PLQY values of the Cs-oleate NCs-in capsules. Also, the likely presence of surface Cs vacancies on the Cs-oleate samples^25^ could facilitate the accommodation of PS moieties on the NCs surface.

### Emission Stability of Capsules over Time and in a Biological Environment

After encapsulation and redispersion in water, we selected a batch of samples with high PLQY and stored the samples in a sealed vial at room temperature and monitored their PLQY for 2 years. Figure 2d displays the evolution of the sample’s PLQY over time. Our protocol proved to be extremely effective to prevent water infiltration through the hydrophobic part of the polymer, and thus, the capsules preserved their emission profile and PLQY while dispersed in water, with values of 60% and 55% PLQY after 12 months for the capsules prepared with Cs-oleate and DDAB-coated CsPbBr_3_ NCs, respectively. That is, the capsules preserve 85% and 68% of their initial PLQY after 1 year in water. This indicates the strong protective character of the polymer shell around the NCs. Photographs of the vials containing the long-aged capsules in water are shown in Figure S14. In parallel, to verify the ability of our capsules to withstand biological media conditions, we monitored their PLQY in saline water (0.9% NaCl solution, see Methods/Experimental). To this aim, we selected the capsules prepared with Cs-oleate coated NCs due to their higher reproducibility in PLQY compared to the DDAB-coated NCs. We found that their emission intensity remains constant over ∼259 days in saline solution (Figure 2e,f). This result hints toward the possible applications of the capsules as PL probes in bioimaging. Therefore, we moved a step further and assessed the cytotoxicity of the capsules by incubating malignant human glioblastoma (U87-MG) cells in a cell culture media enriched with capsules. We assessed first the toxic concentration of Pb and Cs ions (positive controls) on our specific cell line. On the basis of previously reported toxicity data for Pb, a concentration of 600 μg_Pb_/mL causes damage to the ambient and the human body,^36^ and up to date a concentration of 0.1 μg_Pb_/mL is the concern limit for Pb exposure in children.^50^ Therefore, we evaluated the cytotoxicity of Pb and Cs ions administered through our synthesis precursors (Pb(II) acetate trihydrate and Cs carbonate) by initially using two Pb concentrations, 400 and 600 μg/mL. Moreover, considering that the Pb:Cs stoichiometric ratio in our perovskite NCs is equal to 1:1, we also evaluated the toxicity of a mixture of Pb and Cs ions at a 1:1 ratio. In the case of Pb alone, we found that a concentration of 400 μg_Pb_/mL induces a significant drop in U87-MG cell viability from ∼90% at 24 h to 68% at 48 h to 0% at 72 h of incubation (Figure S15a). The viability of cells incubated with the Cs ions instead remained around 80–100% at all the incubated times (Figure S15b), while the drop in cell viability induced by the mixture of Pb and Cs ions showed a similar response to Pb ions, that is, from 85% at 24 h to 70% at 48 h to 0% at 72 h (Figure S15c) at these concentrations. Next, to build a concentration-dependent cytotoxicity curve of Pb on our U87-MG cell line, we focused on Pb ions and the 1:1 mixture of Pb and Cs ions and explored a wide range of concentrations from 0.3 μg_Pb_/mL to values below 400 μg_Pb_/mL with steps of 100 μg_Pb_/mL (Figure S15a,c). We found that a concentration of 300 μg_Pb_/mL already induces a reduction in cell viability of ∼40% after 72 h, whereas when working with lower concentrations (below 200 μg_Pb_/mL), the cell viability remains around 90–100% (Figure S15a). In the case of the 1:1 mixture of Pb and Cs ions at 300 μg_Pb_/mL (and 190 μg_Cs_/mL), the drop in cell viability occurs at earlier times (24 h), see Figure S15c. When the capsule solution at 300 μg_Pb_/mL was administering to the cells, the capsules tended to settle down on the cells (due to the high amount of capsules in the media) and thus induce acute toxicity effects within 24 h of incubation (data not shown). As a note, in this case, to scale up the production, we have used the automated routine repeating the synthesis in 96 vials, a process that overall took less than 90 min.

**Figure 3:**
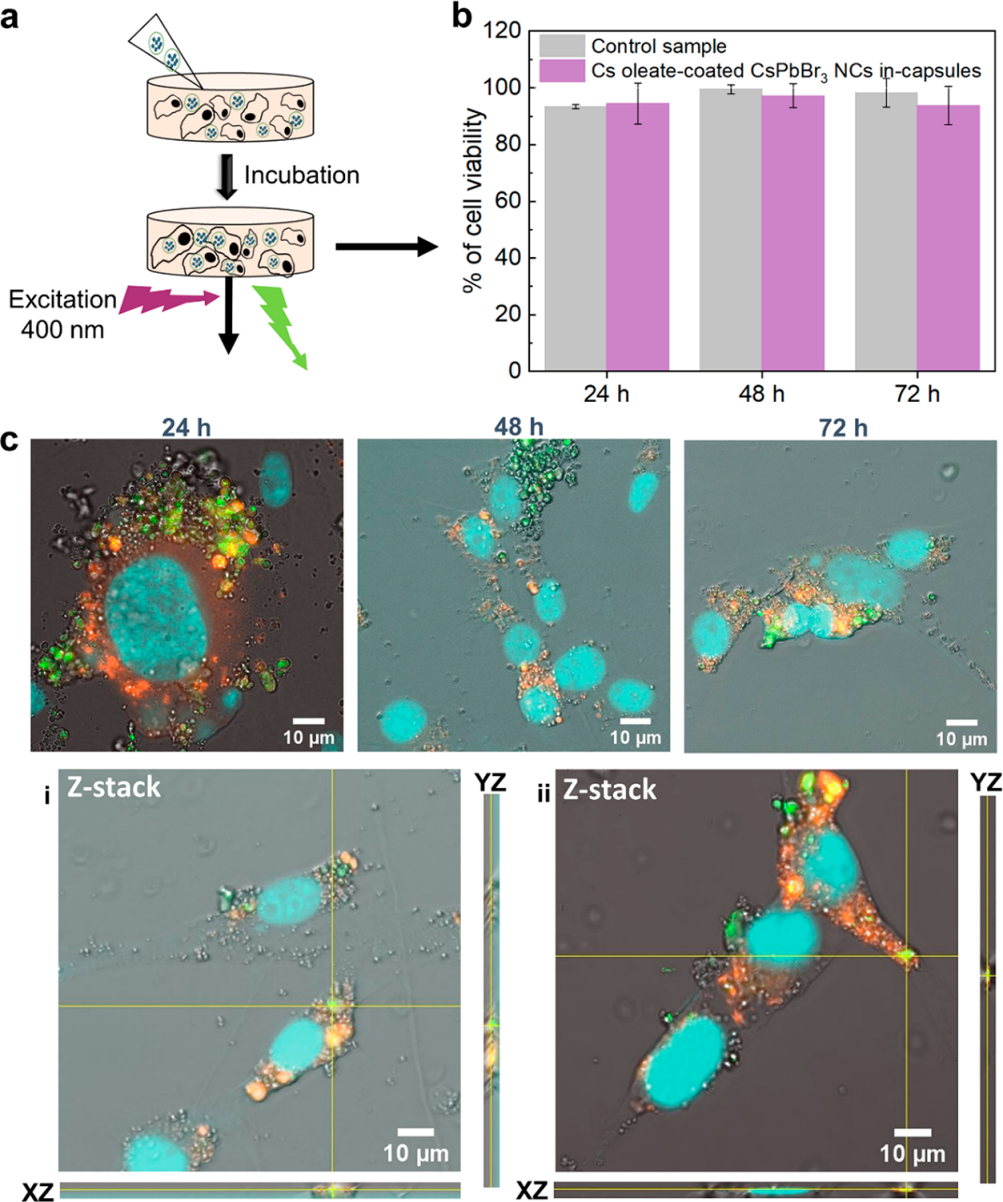
(a) Scheme illustrating the incubation of the cells with capsules for the cell viability and confocal based cell imaging studies. (b) Cell viability study performed by Trypan Blue assay on U87-MG cell line treated with 0.3 μg_Pb_/ml of Cs-oleate coated CsPbBr_3_ NCs in-capsules along 24, 48, and 72 h. Values represent mean ± SD of three independent measurements in two independent experiments. The statistical analysis was performed using one-way ANOVA and multiple comparison Dunn’s test (*p* ≪ 0.05). There is no statistical significance difference between the cell viability data of the control experimental condition and the cells incubated with the capsules. (c) Confocal fluorescent images of cells incubated in a media enriched with Cs-oleate coated CsPbBr_3_ NCs in-capsules for 24, 48, and 72 h. Images were captured at an excitation wavelength of 400 nm. In (c), i and ii images are Z-stack confocal 3D projections collected at (i) low and (ii) high magnification showing the colocalization of capsules (green signal) after 72 h of incubation, with the cell lysosomes (red signal) resulting in yellow spots. Cell nuclei are stained by DAPI dye (blue signal). Scale bars: 10 μm.

To test the maximum capability of our capsules for cell labeling, we selected the lower concentration (0.3 μg_Pb_/mL, see Methods/Experimental), which is also 1000-times lower than the toxicity threshold for Pb and Cs ions (300 μg_Pb_/mL) and incubated the cells over 72 h incubation time (Figure 3a). Like the cytotoxicity test performed by using Pb and Cs ions at 0.3 μg_Pb_/mL, our capsules demonstrated excellent biocompatibility without introducing statistically significant cytotoxicity at this low concentration, as tested by the Trypan Blue assay (Figure 3b). The cell survival percentage at 24, 48, and 72 h of incubation time for the group exposed to the capsules remained like that of the negative control cell groups that were incubated just with media (and did not contain any capsules or Pb or Cs ions). Next, we investigated the uptake of the capsules by the cells at this concentration through fluorescent confocal imaging (Figure 3c and additional images in Figure S16). After capsules exposure for 24, 48, and 72 h and before imaging, the cells were washed and stained with lysotracker for lysosomal staining (in red). Next, the cells were fixed with paraformaldehyde 4% in PBS and stained with 4,6-diamidino-2-phenylindole (DAPI) dye for cell nucleus staining (in blue). The uptake of the capsules was visually evident from their green emission observed after all incubation times when they were excited at a wavelength of 400 nm (Figure 3c). The analysis of the signal for the red channel (for lysosome) and blue one (for nuclei) together with the z-stack 3D projection imaging confirmed the colocalization of capsules (green) within the lysosomal region of the cells (Movies S1 and S2) without reaching the cell nucleus. Additional confocal fluorescent images are displayed in Figure S16. The observed stable emission of our capsules in the acidic lysosomal environment of the cells confirms that they can be exploited for cell/bioimaging, as an alternative to fluorescent dyes,^51,52^ with potential advantages relying on the photostability of these inorganic NCs in water. Also, these results might grant access to the use of such capsules as imaging tools for mapping tumoral tissue where smaller capsules might be desired.^53^ This could be achieved by increasing the injection rate of MeOH as precipitating solvent of the polymer and NCs, in our protocol (Figure S17).

## Conclusions

In summary, we have developed a protocol for the preparation of all inorganic CsPbBr_3_ NCs in water by their encapsulation in a block copolymer. Our study shows that this is a robust approach that is insensitive to the surface coating of the initial NCs and that can be transferred to automated systems, preserving their emission properties in water for over two years with an efficiency of 60%. We further demonstrate that such structural and optical stability is of great benefit for the implementation of these materials in biological media without additional treatments. We showcase this point by using them in toxicity tests that reveal no cytotoxicity, with no cell damage at a dose of 0.3 μg_Pb_/mL. This, in turn, further allows cell inspection by using the capsules as markers at such very low concentration of Pb. We believe that the superior stability of this material system in water coupled to their automated fabrication may enable their transition toward biomedicine and stimulate the exploration of other applications where this combination of features is desirable.

## Methods/Experimental

### Synthesis of CsPbBr_3_ NCs Coated with Two Different Ligands

The NCs were prepared following reported protocols from our group.^43^ Briefly, the Cs-oleate samples (class 1) were prepared by adding 76 mg of lead(II) acetate trihydrate, 16 mg of cesium carbonate, 10 mL of octadecene, and 1.5 mL oleic acid in a 25 mL 3-neck flask (one of the lateral necks is used as a glass finger for a thermocouple and is filled with 0.2 mL of octadecene as a heat transfer medium). Vacuum and magnetic stirring set at 400 rpm were applied until the temperature reached 115 °C. Then 443 mg of didodecylamine, previously dissolved in 1 mL of anhydrous toluene (by heating at 150 °C) was injected into the flask under inert atmosphere. The temperature was decreased to 80 °C after complete dissolution of the metal precursors, and a solution of benzoyl bromide (50 μL) diluted in anhydrous toluene (500 μL) was then swiftly injected into the mixture. The reaction was stopped after 15 s (s) by using a water bath. When the temperature decreased to 40 °C, the mixture was split equally into two 45 mL tubes, and 15 mL of ethyl acetate was added into each tube to destabilize the colloids. Finally, the NCs were collected by centrifugation at 9000 rpm for 20 min, and the precipitate was redispersed in a final volume of 4 mL of toluene. The ligand exchange with didodecyldimethylammonium bromide (DDAB) was carried out to produce the DDAB-coated samples (class 2). Briefly, 3 mL of as-prepared CsPbBr_3_ NCs solution was treated with 2 mL of DDAB dissolved in anhydrous toluene at 0.025 M followed by vigorously stirring for a minute under ambient conditions. Subsequently, 15 mL of ethyl acetate was added into the solution, and then the NCs were collected and redispersed in toluene after centrifugation at 6000 rpm for 10 min. This step was repeated three times by adding the synthesized NCs into a toluene solution containing the DDAB (1 mL, 2 mM) and then washing with 6 mL of ethyl acetate.

The Pb concentration in the synthesized samples was 2.72 and 2.62 mg_Pb_/mL for the Cs-oleate and DDAB-coated NCs, respectively, as measured via ICP-OES, see details below.

### Polymer Encapsulation

The procedure adopted for encapsulating CsPbBr_3_ NCs in polymeric capsules is based on microemulsion self-assembling techniques.^46,54,55^ To a 4 mL glass vial with sept-cap, 100 μL of polystyrene-*block*-poly(acrylic acid) (PS-*b*-PAA) with a molecular weight of 29:5 kDa (32 mg in 1 mL toluene/THF mixture (9:1 in volume)) and 25 μL of the NC solution in toluene were added. The mixture was sonicated for 5 min at room temperature. After orbital shaking for additional 15 min at 1000 rpm, 150 μL of fresh MeOH was injected with a syringe pump at a rate of 250 μL/min under shaking. After 30 s, an additional 400 μL of MeOH was added manually (one-shot) under shaking at a rate of 600 rpm. Without shaking, 250 μL of hexane was then added to the resulting dispersion, which was next centrifuged at 6000 rpm for 10 min. The supernatant was discarded, and the pellet was redispersed in 300 μL of fresh MeOH and the vial sonicated for 30 min. The capsules were precipitated once more via hexane addition (250 μL) and centrifugation (6000 rpm for 10 min) and finally redispersed in 1.2 mL of Milli-Q-water via sonication (30 min) and shaking overnight at 1000 rpm before further analysis. The concentration of Pb on the prepared samples was 0.17 and 0.1 mg_Pb_/mL for the Cs-oleate and DDAB-coated NCs, respectively, as measured via ICP-OES, see details below.

### Automation of CsPbBr_3_-Based Capsules Fabrication in Water

The manual fabrication process of encapsulated NCs was translated into an automated fabrication, with minor modifications, using the commercial Microlab NIMBUS4 (Hamilton) liquid handling robotic system equipped with four independent micropipettes arms. The reaction protocol was programmed in Hamilton Method Editor software to parallel the manual approach. All necessary reagents for the reaction were prefilled in solution holders and placed in a specific position on the robot operation deck (Figure S7, right panel), following the same order of addition and mixing of the reagents as in the manual protocol. The reaction took place in 8 mL glass vials loaded into a 24-well aluminum plate and placed on a Hamilton Heater Shaker (HHS) module on the NIMBUS4 deck. A complete workflow sketch is displayed in Figure S6.

### Structural and Morphological Characterization

The assessment of the capsule’s morphology and size was performed by using a JEOL JEM-1400 operating at 120 kV. The samples were prepared by drop-casting the water dispersions on carbon-coated copper grids previously treated with oxygen plasma. Mean hydrodynamic size and Zeta-potential (average of three consequent measurements) of the Cs-oleate coated CsPbBr_3_ NCs in-capsules diluted in aqueous solution were measured using a dynamic light scattering (DLS) system (Zetasizer Nano ZS90 (Malvern, UK)) with He–Ne laser (4.0 mW) of 633 nm and a photodiode detector. The NC concentration was measured based on their Pb content for both the initial NCs in toluene and capsules dispersed in water via ICP-OES by using a Thermo Fisher iCAP 7600 DUO instrument. For the NCs, 50 μL was digested overnight in a mixture of 200 μL of H_2_O_2_ nitric acid and diluted in deionized water for a total volume of 10 mL in a calibrated flask. For the NCs in-capsules, two batches of prepared samples in water (1.2 mL) were concentrated in 100 μL of Milli-Q water and digested overnight in a mixture of 100 μL of HNO_3_ tracemetal degree and diluted in deionized water for a total volume of 10 mL in a calibrated flask. All the suspensions were filtered before analysis by using PTFE filters. X-ray diffraction patterns were collected from the NCs and the capsules on a PANalytical Empyrean X-ray diffractometer operated at 45 kV and 40 mA and equipped with a 1.8 kW CuKα ceramic X-ray tube. Samples were prepared by drop casting 5 μL of each capsule solution at a concentration of 200 μg_Pb_/mL on a zero-diffraction substrate.

### Optical Characterization

Photoluminescence spectra were collected from the solutions containing starting NCs as well as from capsules dispersed in water, ethanol, and MeOH using a Varian Cary Eclipse fluorescence spectrophotometer. The samples were excited at 350 nm using a xenon lamp source. The samples in ethanol and MeOH showed in Figure S4 were prepared by using 50 μL of the capsules in water followed by removal of water and addition of 1 mL of the respective polar solvent. Steady-state absorbance spectra were collected on a Varian Cary 5000 ultraviolet–visible–near-infrared (UV–vis–NIR) spectrophotometer equipped with an external diffuse reflectance accessory and operating in absorption geometry. The samples were prepared from drop casting an aliquot of 100 μL from the capsules in water and drop casting it on a quartz substrate.

The PLQY of the samples was measured using an Edinburgh Instruments (FLS920) fluorescence spectrometer equipped with an integrating sphere. The samples were excited at 400 nm using a xenon lamp source. The dispersion of empty capsules (prepared without NCs following the same fabrication steps of the capsules with NCs) were used as the reference blank. Light absorption due to scattering inside the sphere was taken into account for the PLQY calculation by collecting three different spectra: (1) directly exciting the sample in the sphere, (2) indirectly exciting sample in the sphere, and (3) with the reference blank in the sphere in the direct excitation position. Three measurements were performed for each sample.

The PL profile and the PL intensity over time (for max 259 days) of the capsules in saline solution were tested by diluting 500 μL of the capsules (from the 1.2 mL of capsules in water prepared in a single reaction) in 500 μL of 1.8% NaCl solutions. The final volume corresponds to 0.9% NaCl.

### Cell Cultures

Human brain glioblastoma cells (U87-MG, donated by Dr. Emilio Ciusani from Carlo Besta Neurological Institute from Milano) were maintained in Dulbecco’s modified Eagle’s medium high glucose (D5671, Sigma-Aldrich) supplemented with 10% heat-inactivated fetal bovine serum (Sigma-Aldrich, F4135), 2 mM l-glutamine, and 100 U/mL penicillin/streptomycin (Sigma-Aldrich P4333). The cell line was maintained at 37 °C in 5% CO_2_ and 95% air in a humidified atmosphere and passaged twice a week when reaching a confluence of ∼80–90%.

### Cell Viability Study

To evaluate the biocompatibility of the Cs-oleate coated NCs in-capsules, cell viability was measured by Trypan Blue assay following incubation of the U87-MG cells at different concentrations for 24, 48, and 72 h. The amount of capsules added to the media was based on the Pb content as determined by ICP and fixed at 0.3 μg_Pb_/mL or at 300 μg_Pb_/mL in 500 μL of complete cell culture media. The toxicity of Pb, Cs, and Pb:Cs mixed ions (at 1:1 stoichiometric of Pb and Cs) was evaluated by Presto Blue assay. Pb and Cs mother solutions in Milli-Q water were prepared separately by using the Pb and Cs precursors in the synthesis of the Cs-oleate coated NCs (Pb(II) acetate trihydrate and Cs carbonate). The content of Pb in the Pb(II) acetate trihydrate mother solution was 3.40 mg_Pb_/mL, as measured by ICP. The Cs mother solution was prepared by dissolving Cs carbonate in Milli-Q water to achieve a stoichiometrically equivalent amount of Cs with respect to Pb corresponding to 2.16 mg_Cs_/mL. For the toxicity tests shown in Figure S15, dilutions were prepared from these mother solutions by using complete cell culture media to achieve a final volume of 500 μL per well and reach the different concentrations of Pb and Cs ions to be tested, within a range from 0.3 μg/mL to 600 μg/mL. The choice of the lower capsule concentration was based on previous work where the authors studied a range from 0.01 to 25 μg/mL of beads.^56^ For cell viability assays, cells were seeded into 24-well plates at different confluencies (cell viability at 24 h: 100 000 cells/well; cell viability at 48 h: 50 000 cells/well; cell viability at 72 h: 25 000 cells/well). Twenty-four hours after cell seeding, capsules or ion solutions at the previously specified concentrations were added to the wells containing fresh media. In the case of the cells incubated with capsules, after the respective incubation times, the cell culture medium was removed and the wells were gently washed with the phosphate-buffered saline (PBS 1X pH = 7.4) medium. Cells were detached with Trypsin-EDTA (0.25%, Sigma-Aldrich) at 37 °C for 5 min followed by the addition of Dulbecco’s modified Eagle’s medium (DMEM) supplemented with 10% heat-inactivated fetal bovine serum to block the trypsin. Upon centrifugation at 1000 rpm for 5 min, the cell pellet recovered after removal of the medium was resuspended in 500 μL of cell culture medium and diluted 1:1 in volume with an aqueous solution of Trypan Blue 0.4% (Sigma-Aldrich T8154), and live (colorless) or dead (blue) cells were counted by hemocytometer under the microscope. In the case of the cells incubated with the ion solutions (Figure S15), after the respective incubation times, the cell culture medium was removed and a solution of 10% (volume) of Presto Blue (ThermoFisher Scientific) in a final volume of 300 μL of PBS 1X buffer was added to each well. Next, the plates were incubated at 37 °C for 1 h. After this, 300 μL of each well was collected and distributed in 3 wells with each 100 μL in a p96 multiwell plate. Reading of each well was performed by using a multiwell spectrophotometer (Cary 50) using multiple wavelength (570–600 nm).

### Confocal Imaging

U87-MG cells were seeded on 14 mm glass coverslips. Cells were incubated with 0.3 μg_Pb_/ml of capsules, at different time points of 24, 48, and 72 h. Before imaging, cells were washed with PBS 1X pH = 7.4 to remove not uptaken capsules in the media, stained using a lysotracker red (50 nM) (LysoTracker Red DND-99, Thermo Fisher Scientific) in cell culture media incubated at 37 °C for 45 min. After lysotracker staining, for cell nuclei staining, cells were fixed with paraformaldehyde 4% in PBS for 20 min at room temperature, washed three times with PBS 1X pH = 7.4, and stained with DAPI (Thermo Fisher Scientific) nuclei staining (1:500) (ratio DAPI versus final volume of media) in PBS-0.25% Triton X-100 for 20 min. After washing with PBS 1X pH = 7.4, samples were mounted with Prolong Gold Antifade DAPI (a protective mounting agent that protects fluorescence) and dried at room temperature. To image the desired green fluorescence of capsules after cell fixation by confocal microscopy, the cells under confocal laser were excited at 400 nm. For cell nuclei, DAPI staining cells were excited at 359 nm and for lysotracker red, cells were excited at 576 nm, respectively.

## Supporting Information Available

Comparison among reported materials; TEM, ICP analysis, DLS, PL, cytotoxicity test results for Pb ions, Cs ions, and 1:1 mixture of Pb and Cs ions; additional confocal fluorescent images; photographs of fresh emitting capsules prepared by robot and on-bench, detailed description of automated protocol, PLQY data comparison between samples prepared by robot and by human (PDF)
Movie S1 (AVI): Z-stack orthogonal confocal 3D projection showing the co-localized capsules within the cell lysozyme.
Movie S2 (AVI): Z-stack orthogonal confocal 3D projection of another region showing the co-localized capsules within the lysozyme.

## Supplementary Material

nn2c01556_si_001.pdf

nn2c01556_si_002.avi

nn2c01556_si_003.avi
