# Supplementary material for: Highly Emitting Perovskite Nanocrystals with 2-Year Stability in Water through an Automated Polymer Encapsulation for Bioimaging
Source: ACS Nano. 2022 Aug 1;16(9):13657–66. doi: 10.1021/acsnano.2c01556 (PMC9527756; doi:10.1021/acsnano.2c01556)
Supplement: Supplementary file 1 — nn2c01556_si_001.pdf [file nn2c01556_si_001.pdf]

Supporting Information

Highly Emitting Perovskite Nanocrystals with 2-Year Stability in Water through an Automated Polymer Encapsulation for Bioimaging

Sahitya Kumar Avugadda, Andrea Castelli, Balaji Dhanabalan, Tamara Fernandez, Niccolo Silvestri, Cynthia Collantes, Dmitry Baranov, Muhammad Imran, Liberato Manna, Teresa Pellegrino, and Milena P. Arciniegas**

*Emails: Teresa.Pellegrino@iit.it and Milena.Arciniegas@iit.it

Table S1. Recent reported works on enhancing the stability of all-inorganic perovskite nanocrystals in water by different approaches. Note that only the first four works targeted a bioapplication. NI: no investigated.

Material	Reported stability in water over time	Application	Reference
$CsPbBr_3$ NCs-in capsules	2 years	Bioimaging	This work
$CsPbBr_3/SiO_2$/mPEG-DSPE	5 days	Multiphoton bioimaging	1
$CsPbBr_3/SiO_2$-C_{18}-PC Core–Shell	10 days	Cell imaging	2
$CsPbBr_3$/PBMA nanocomposites	7 days	High contrast X-ray imaging	3
PEG–PPG-PEG $CsPbBr_3$ polymer encapsulation	8 days	Tumor-Derived Exosome Imaging	4
Polyacrylate polymer-grafted CsPbBr3 NCs	7 days	NI	5
$MAPbBr_3$ polymer nanocomposite films	18 months	NI	6
$CsPbBr_3$–$CsPb_2Br_5$ – PMMA	40 days	Colour converters	7
$CsPbBr_3$/PMSQ	14 days	LED	8
$CsPbBr_3$/ZnS Core/Shell	2 days	PL life time	9
$CsPbBr_3/SiO_2$	42 days	2-photon lasing	10
Pb-poor synthesis of $CsPbBr_3$	200 days	Electrocatalysis	11
$CsPbBr_3/Cs_4PbBr_6$	8 days	NI	12
$CsPbBr_3$ quantum dots -loaded polymeric nanospheres	8 days	NI	13

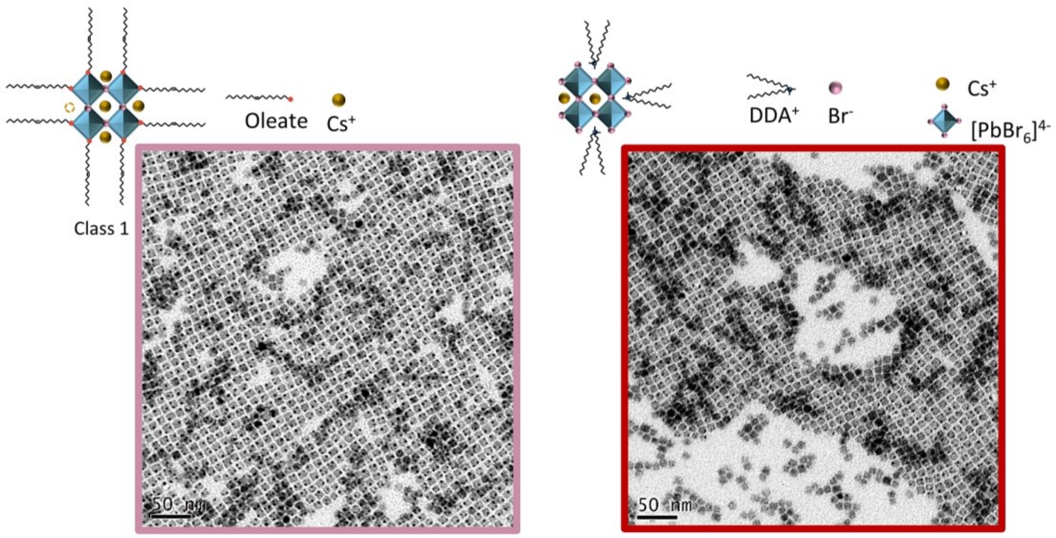

Figure S1. TEM images collected from drop-casted solutions of Cs-oleate (left) and DDAB-coated (right) $CsPbBr_3$ NCs in toluene on Cu-grids prior formation of the capsules. The embedded cartoons highlight the structure of the NCs coated with Cs-oleate (class 1) and didodecyl dimethylammonium (DDAB) (class 2) ligands.

Table S2. Concentration of both initial NCs in toluene and capsules dispersed in water The values refer to the Pb content on all the samples. The analysis was performed via ICP. Note that the values obtained from the capsules were estimated by concentrating two batches of samples of 1.2 mL in 100 μl.

Sample	Concentration [mg_{Pb}/mL]
Cs-oleate coated $CsPbBr_3$ NCs	2.72
DDAB-coated $CsPbBr_3$ NCs	2.62
Cs-oleate coated $CsPbBr_3$ NCs in-capsules	0.17
DDAB-coated $CsPbBr_3$ NCs in-capsules	0.10

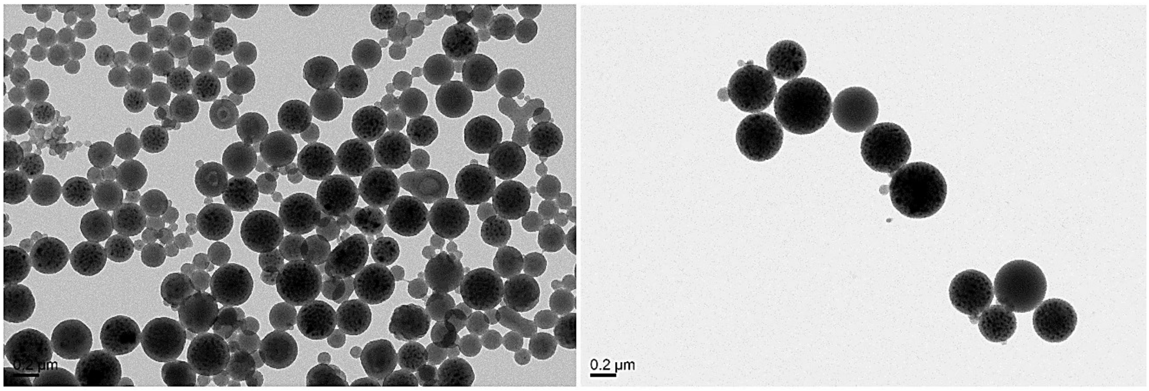

Figure S2. TEM images collected at low magnification from dried DDAB-coated $CsPbBr_3$ capsules deposited from water dispersions on Cu TEM grids. Scale bars: 0.2 μm.

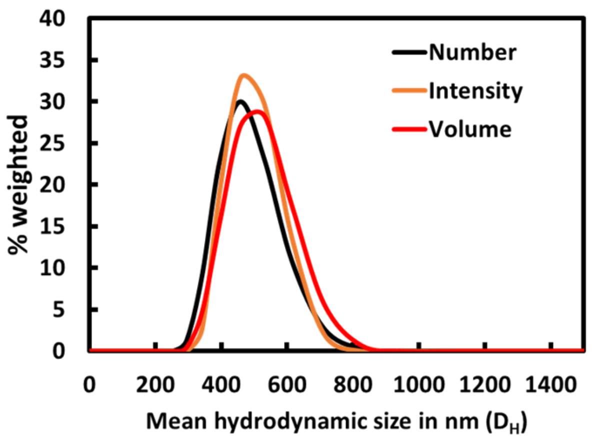

	D_H by Intensity (nm)	D_H by Number (nm)	D_H by Volume (nm)	PDI
Cs-oleate NCs in-capsules	491±80	473±90	507±96	0.66

Figure S3. The mean hydrodynamic size, D_H, of Cs-oleate coated $CsPbBr_3$ NCs in-capsules dispersed in water. The standard deviation values presented were calculated from the full-width half maxima (FWHM) of the peaks.

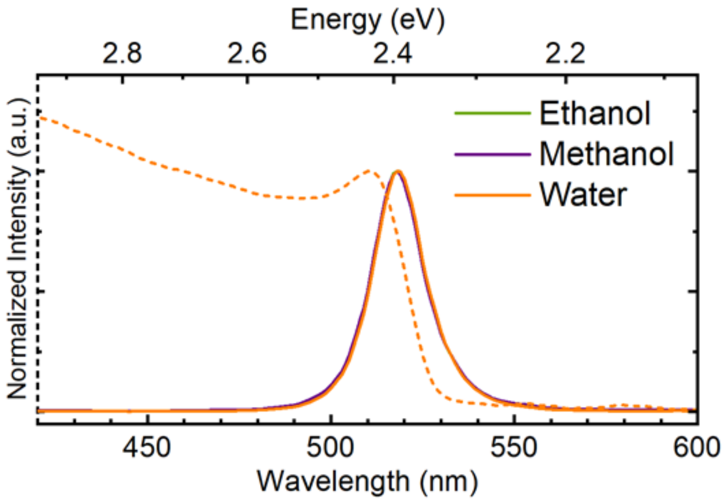

Figure S4. PL spectra collected from the prepared Cs-oleate coated $CsPbBr_3$ capsules dispersed in different polar solvents after 18 months of their fabrication denoting the robustness of the capsules. Excitation wavelength: 350 nm. The absorbance spectrum of the capsules is shown in orange dotted line and it was collected from a drop casted dispersion of fresh capsules in water.

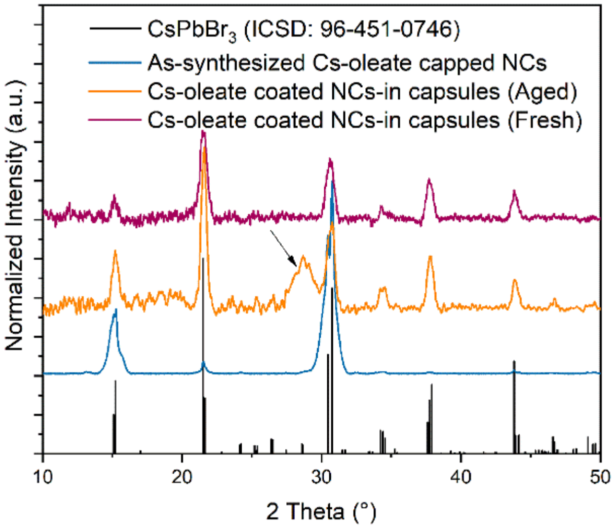

Figure S5. X-ray diffraction patterns collected from the as-synthesized Cs-oleate coated NCs, both fresh and 24 months aged samples of capsules dispersed in water, and the reference pattern (COD 96-451-0746). The arrow indicates the remaining signal from the amorphous polymer.

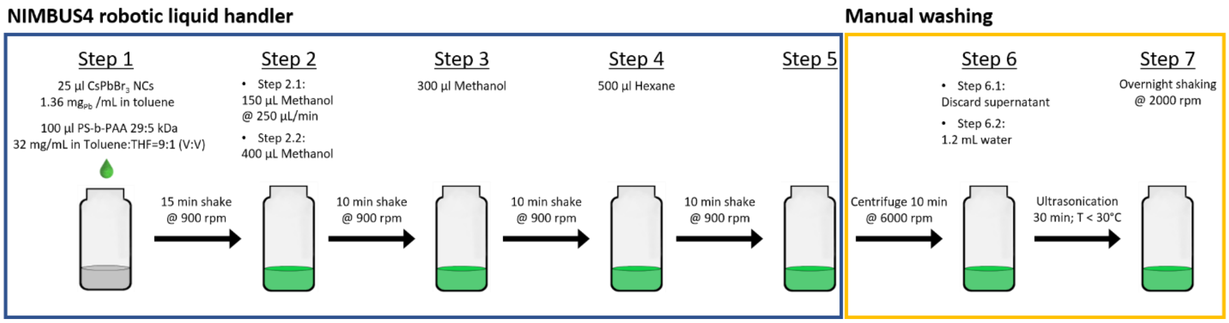

Figure S6. Workflow developed to approach the fabrication of $CsPbBr_3$ NCs-in polymer capsules through a robotic arm. The blue frame higlights the steps performed by Nimbus4 while the orange one the steps performed by a human hand.

Step-by-step procedure for NIMBUS4 robotic liquid handler

1. The polymer and NCs in solution were manually prepared separately following the developed bench procedure. Solution 1 is a mixture of 25 μL of $CsPbBr_3$ NCs (1.36 mg_{Pb}/mL) in toluene and 100 μL of PS-*b*-PAA 32 mg/mL in a toluene:THF solvent mixture (9:1 vol). Reagent 1 and Reagent 2 are pure solvents methanol and hexane, respectively. All reagents and solutions were prepared in 8 mL glass vials and manually loaded into the designated position on robot deck. The 24 glass vials pre-filled with Solution 1 were used as reaction pots and placed in a 24-well aluminium rack on the Hamilton Heater Shaker (HHS) module. Sets of four glass vials of 8 ml containing Reagents 1 and 2 were placed in a 24-well polypropylene rack (Step 1). Unless specified, the liquid aspiration and dispensing parameters were kept as detailed for each liquid class by the producer.

2. The automated procedure initiated by orbital shaking at 900 rpm and pre-heating the aluminium rack to a fixed temperature of 25°C. The shaking speed and temperature were kept for the entire procedure. The actual temperature of the HHS module was measured and recorded by the instrument's software.

3. After the temperature of the HHS stabilized at 25 ºC, the robot started to dispenses 150 µL of Reagent 1 into each of the 24 (8-mL) glass vials located in the aluminum rack (containing Solution 1) at a flow rate of 250 µL/min (Step 2.1). This first addition of Reagent 1 was immediately followed by a second addition of Reagent 1 (400 µL, Step 2.2). Once completed and after additional 10 minutes of shaking, a third addition of Reagent 1 (300 µL, Step 3) was performed by the liquid handler, followed by 10 minutes of shaking. The liquid dispensing process is programmed and automatically carried out.

4. After the last addition of Reagent 1, 500 µl of Reagent 2 was dispensed into the 24 glass vials followed by 10 minutes of shaking (Step 4).

5. After the last shaking step, the robotic procedure was stopped and an operator removed the vials from the reaction rack and visually inspected each vial (Step 5) for controlling the appearance and volume of the final product, performing a washing step and re-suspending the NCs in water (Step 6 and 7) before evaluating their PLQY, as described in the bench protocol.

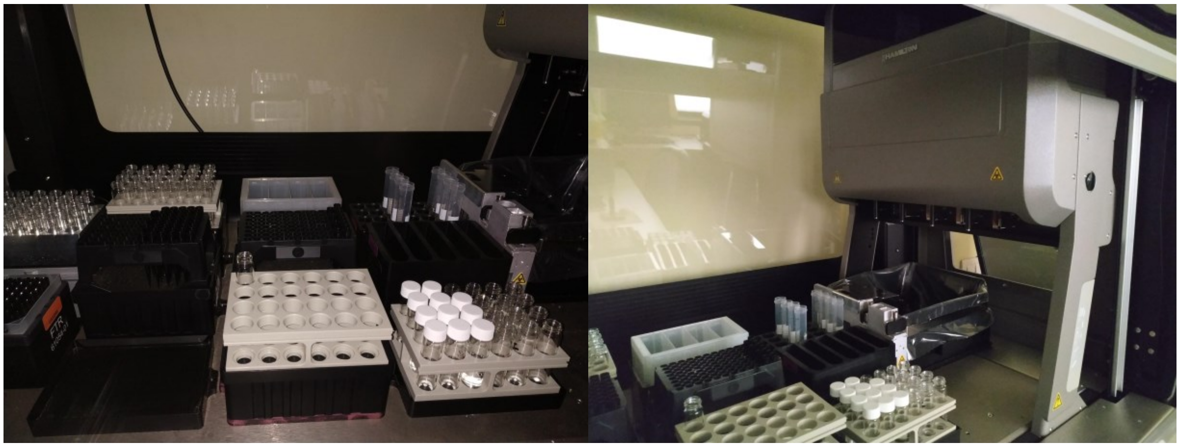

Figure S7. Photograph showing the NIMBUS platform used for the capsule's fabrication (left) and the robotic arm (right).

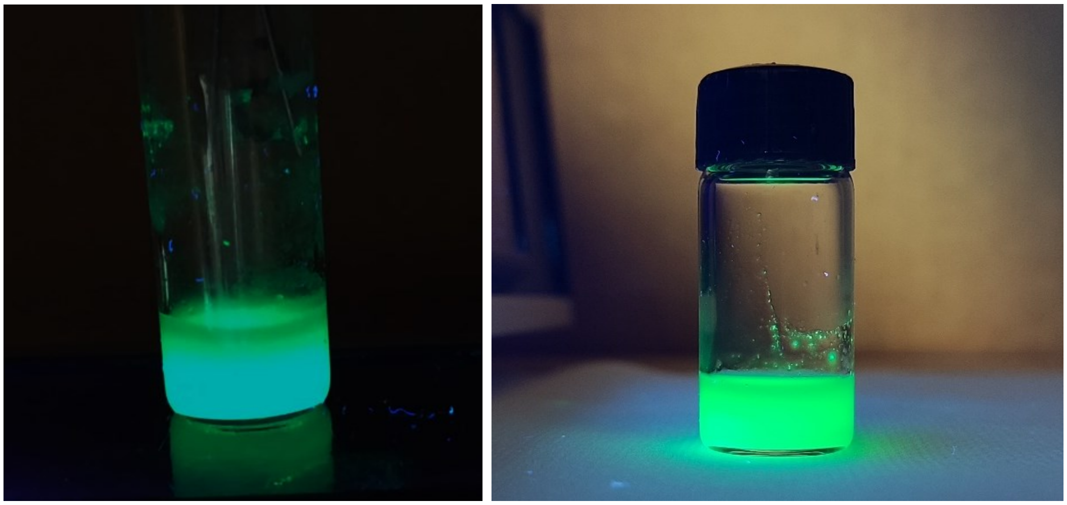

Figure S8. Picture of freshly fabricated DDAB-coated (left) and Cs-oleate (right) $CsPbBr_3$ NCs in-capsules dispersed in water and prepared with Nimbus robotic arm.

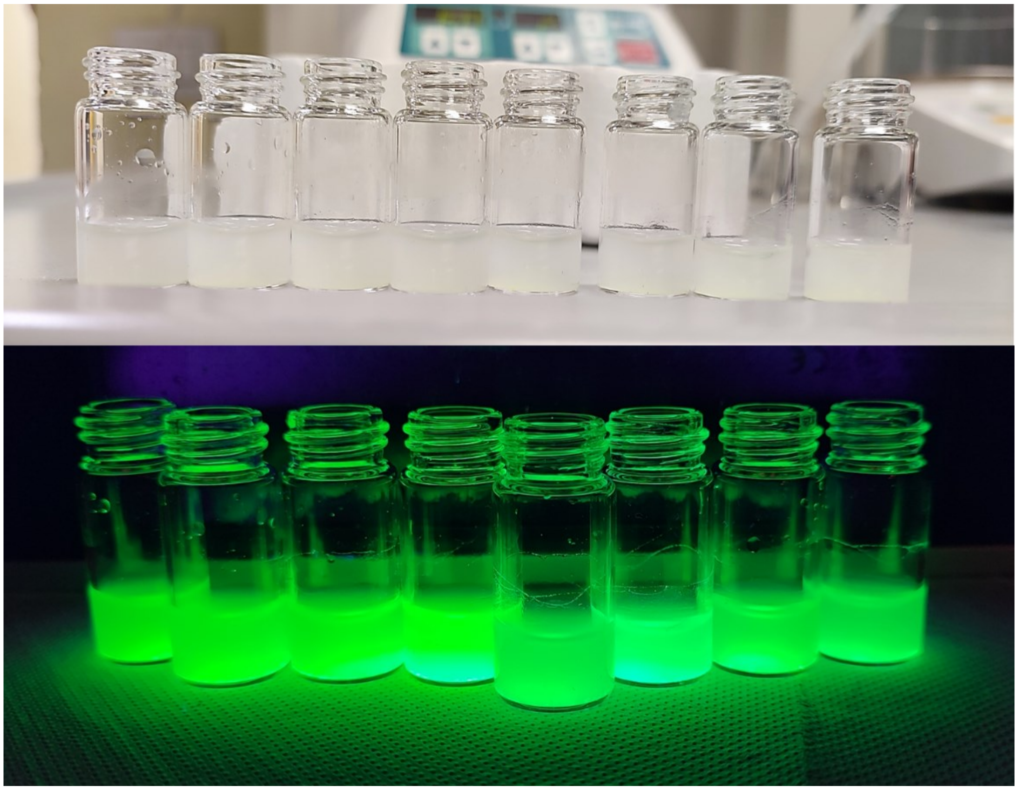

Figure S9. Picture showing a set of eight vials containing Cs-oleate coated $CsPbBr_3$ NCs in-capsules dispersed in water and fabricated in parallel with Nimbus robotic arm under normal light (top) and UV light (bottom).

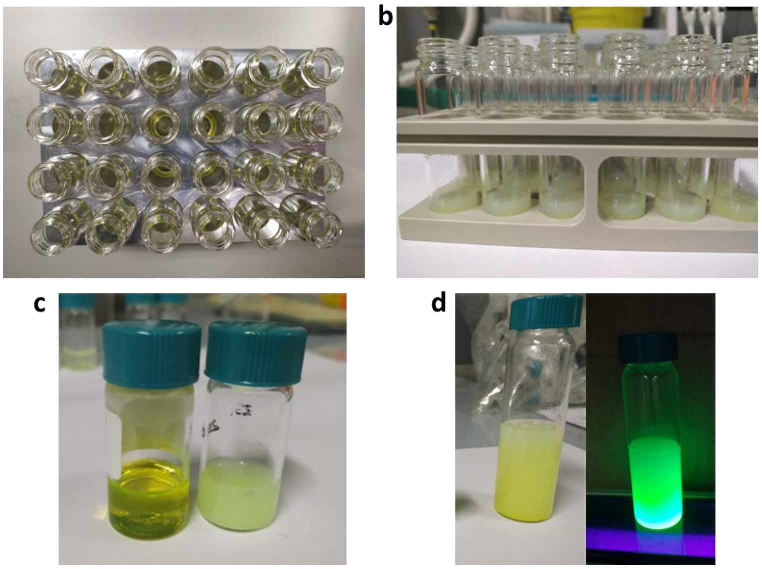

Figure S10. a-b, Photographs of a batch of 24 vials before and after the formation of the capsules. c, Photograph of the freshly synthesized sample of Cs-oleate NCs and the corresponding capsules prepared the same day following the on-bench protocol. d, photograph of 96 samples prepared with Nimbus and concentrated in 2 mL of MilliQ water under normal light and UV lamp.

Table S3. PLQY measured from a set of eight samples of Cs-oleate coated $CsPbBr_3$ NCs in-capsules dispersed in water and fabricated in parallel with Nimbus robotic arm. PLQY average value 55.4 ± 6.0 %. The last column shows the collected values from different samples prepared on-bench by a human.

Sample track number	PLQY [%] Robotic arm	Sample track number	PLQY [%] Human
NS_R1	57	NS_H1	61
NS_R2	45	NS_H2	46
NS_R3	60	NS_H3	51
NS_R4	59	NS_H4	62
NS_R5	56	NS_H5	53
NS_R6	52	-	-
NS_R7	50	-	-
NS_R8	64	-	-

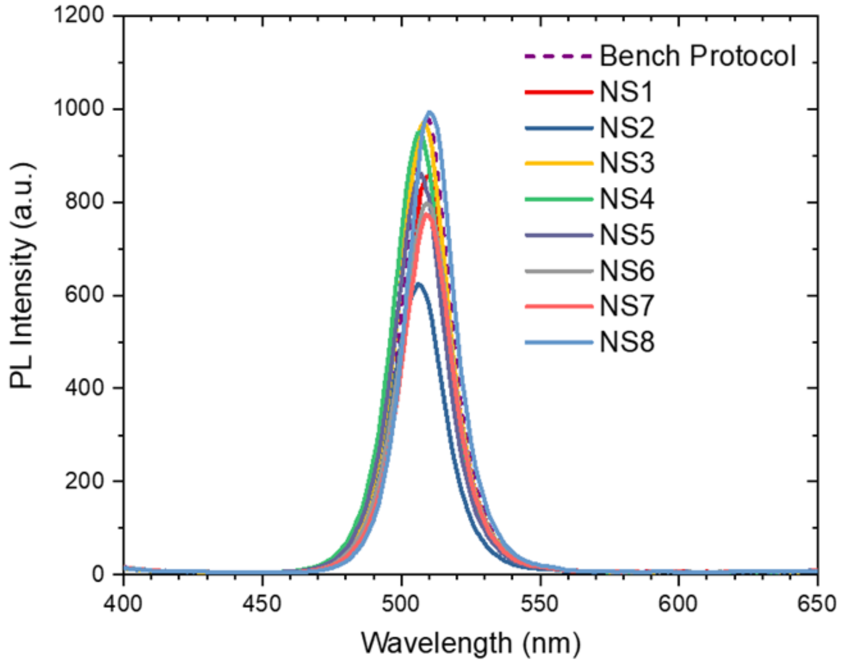

Figure S11. PL spectra collected from the set of samples prepared with the robotic arm and listed in Table S2.

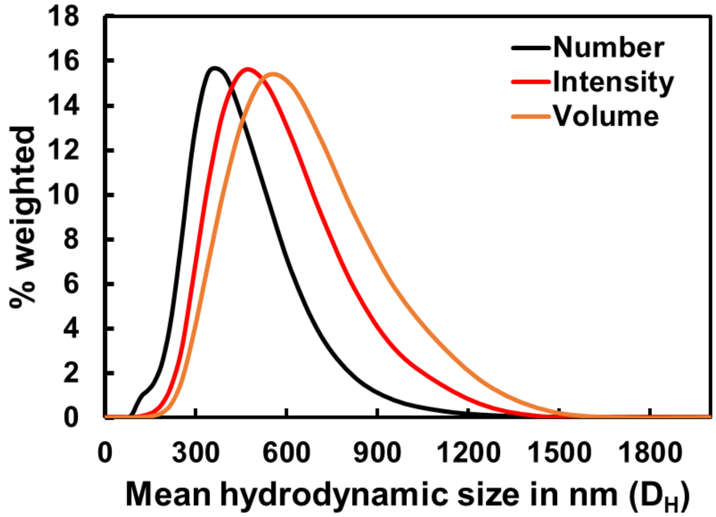

	D_H by Intensity (nm)	D_H by Number (nm)	D_H by Volume (nm)	PDI
Cs-oleate NCs in-capsules	516±193	401±161	594±223	0.18

Figure S12. The mean hydrodynamic size, D_H, of Cs-oleate coated $CsPbBr_3$ NCs in-capsules dispersed in water and prepared with the robotic arm. The standard deviation values presented were calculated from the full-width half maxima (FWHM) of the peaks. The Zeta-potential measurements resulted in a value of -37 ± 3 mV.

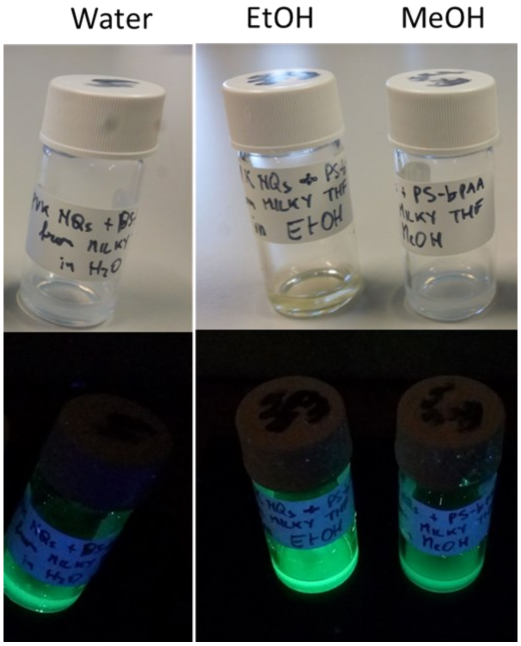

Figure S13. Photographs of the Cs-oleate coated $CsPbBr_3$ NCs in-capsules dispersed in different polar solvents taken under normal light (top) and UV light (bottom). The pictures were collected after preserving the capsules for two weeks in the corresponding solvent. The samples were fabricated as described in the main text and aliquots of the fresh samples were redispersed in the different solvents.

Table S4. PLQY values measured from different batches of capsules prepared following the bench protocol. The estimated variance (s^2) between PLQY values obtained from the capsules prepared with Cs-oleate NCs is narrower (s^2 = 37) compared to that of the DDAB NCs-in capsules (s2 = 143).

PLQY [%]	
**Cs-oleate Samples**	**DDAB samples**
61	65
46	42
51	70
62	55
53	65
	41
	39
	65

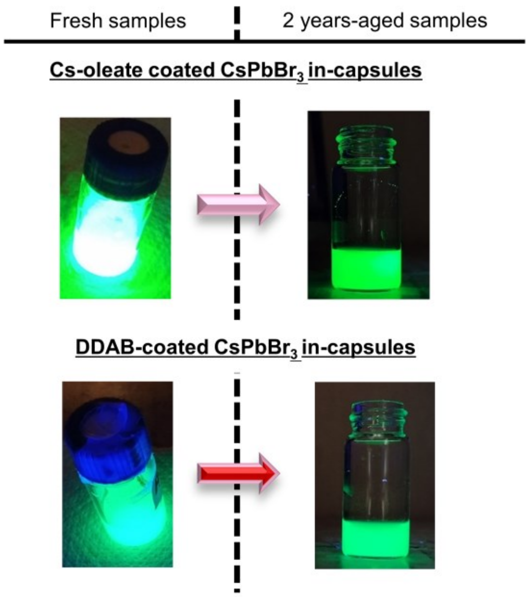

Figure S14. Photographs of the vials containing the capsules in water under UV light. The pictures were taken immediately after their fabrication (fresh samples on the left) and after 18 and 24 months (aged samples on the right) from the same solutions of Cs-oleate and DDAB NCs in-capsules, respectively.

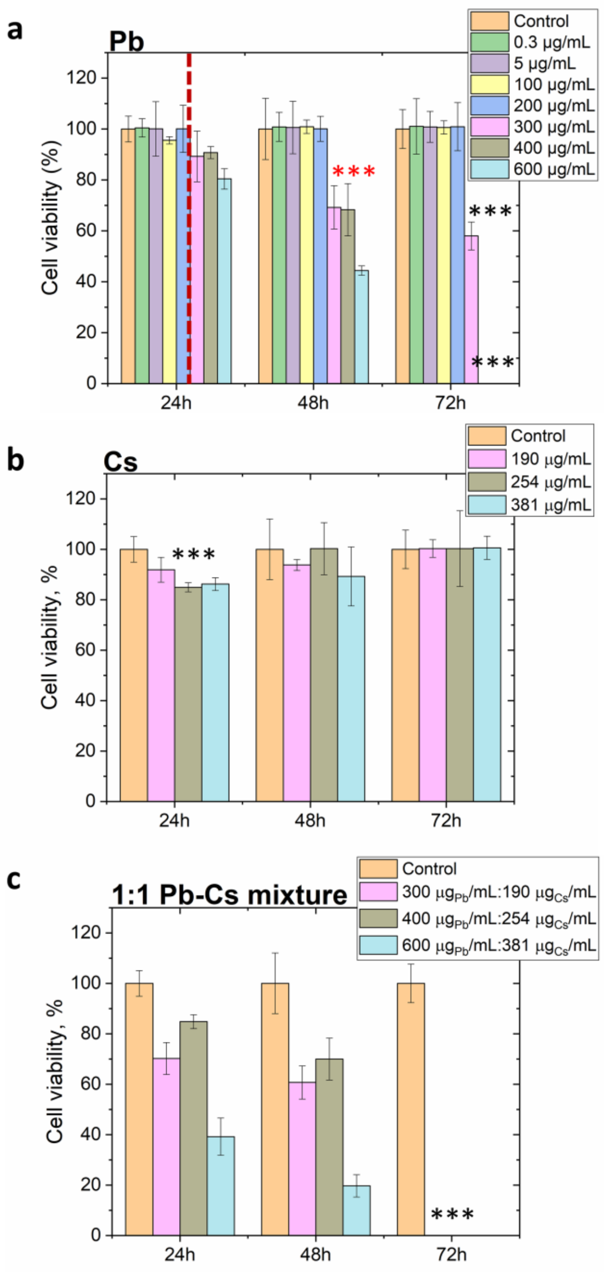

Figure S15. Cell viability study performed by Presto Blue assay on U87 cell line treated with different concentration of (a) Pb ions, (b) Cs ions, and (c) 1:1 mixture of Pb and Cs ions in MilliQ water along 24 h, 48 h and 72 h. Values represent mean with bars indicating the standard deviation (SD) calculated from three independent experiments. The red dotted line in (a) highlights the concentration threshold (300 μg_{Pb}/mL) from which a significant reduction in cell viability is observed at 48 h. The missing columns for each time mean 0 % cell viability. Statistical analysis was performed using one-way ANOVA with a Dunn´s post hoc test. ***p = <0,001, indicating statistically significant differences. In (a) red asterisks represent statistical differences in cell viability at 48 h with respect to 24 h of incubation; black asterisks in (a) and (c) represent statistical differences of 72 h respect to 24 h. In (b) black asterisks represent statistical differences of 24h respect to 72 h.

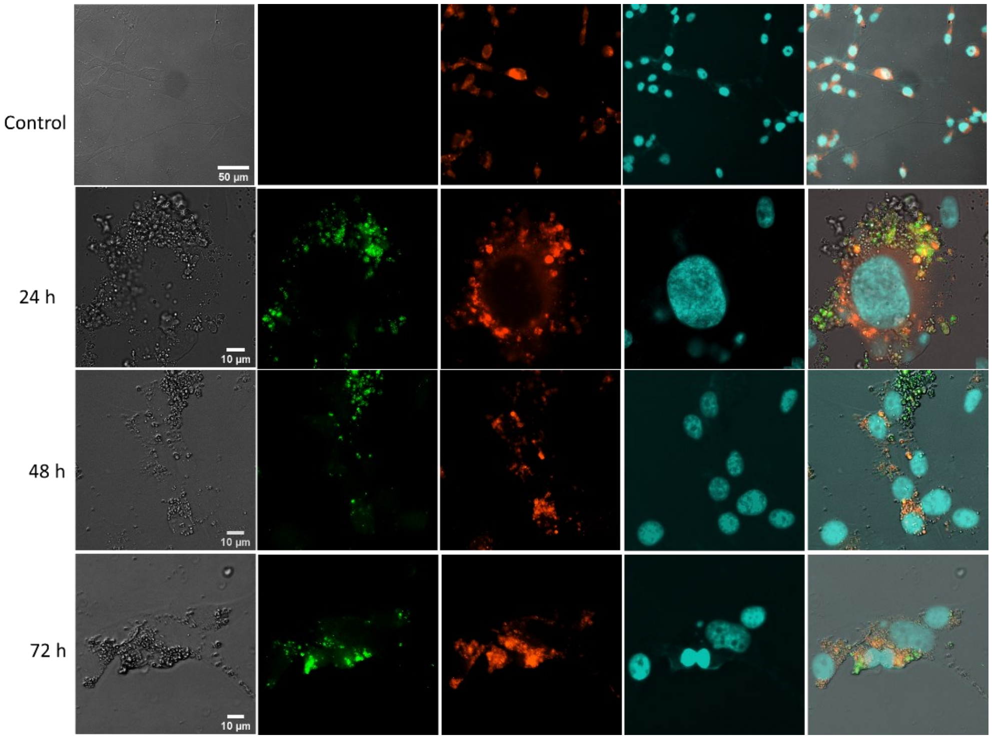

Figure S16. Additional confocal fluorescent images of U87 cells incubated in capsules rich media for 24, 48, and 72 h in comparison to the control cells incubated in media free of capsules. Scale bars: 10 µm. Green signal is provided by capsules, red signal is provided by lysotracker and blue signal is due to DAPI dye.

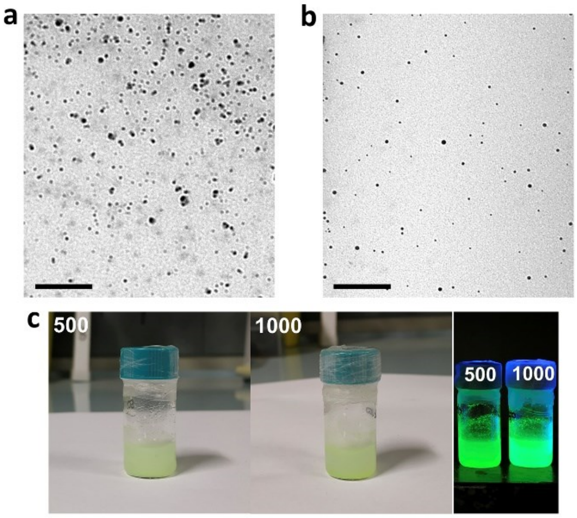

Figure S17. a-b, TEM images collected at low magnification from dried Cs oleate-coated $CsPbBr_3$ capsules prepared by adding methanol at two different flow rates, (a) 500 µl/min and (b) 1000 µl/min and deposited from water dispersions on Cu TEM grids. Scale bars: 0.2 µm. c, Photographs of the capsule solutions prepared at 500 and 1000 µl/min under daylight (left panels) and under UV light (right panel). We observed three size populations from batches of capsules prepared at

500 µl/min, from 20 nm to 150 nm, while in the case of capsules prepared at 1000 µl/min we observed two populations, from 10 nm to 60 nm. Further cleaning may help in the size selection and removal of empty capsules.

Supplementary Movies

Movie S1 and Movie S2. Z-stack orthogonal confocal 3D projection showing the co-localized capsules within the cell lysozyme.

References

1. Chan, K. K.; Giovanni, D.; He, H.; Sum, T. C.; Yong, K.-T., Water-Stable All-Inorganic Perovskite Nanocrystals with Nonlinear Optical Properties for Targeted Multiphoton Bioimaging. *ACS Appl. Nano Mater.* **2021,** *4* (9), 9022-9033.
2. Wu, H.; Chen, Y.; Zhang, W.; Khan, M. S.; Chi, Y., Water-Dispersed Perovskite Nanocube@SiO2-C18-PC Core–Shell Nanoparticles for Cell Imaging. *ACS Appl. Nano Mater.* **2021,** *4* (11), 11791–11800.
3. Nie, J.; Li, C.; Zhou, S.; Huang, J.; Ouyang, X.; Xu, Q., High Photoluminescence Quantum Yield Perovskite/Polymer Nanocomposites for High Contrast X-ray Imaging. *ACS Appl. Mater. Interfaces* **2021**.
4. Pramanik, A.; Gates, K.; Patibandla, S.; Davis, D.; Begum, S.; Iftekhar, R.; Alamgir, S.; Paige, S.; Porter, M. M.; Ray, P. C., Water-Soluble and Bright Luminescent Cesium–Lead–Bromide Perovskite Quantum Dot–Polymer Composites for Tumor-Derived Exosome Imaging. *ACS Appl. Bio Mater.* **2019,** *2* (12), 5872-5879.
5. Jin, X.; Ma, K.; Chakkamalayath, J.; Morsby, J.; Gao, H., In Situ Photocatalyzed Polymerization to Stabilize Perovskite Nanocrystals in Protic Solvents. *ACS Energy Lett.* **2022,** *7*, 610-616.
6. Carrizo, A. F.; Belmonte, G. K.; Santos, F. S.; Backes, C. W.; B. Strapasson, G.; Schmidt, L. C.; Rodembusch, F. S.; Weibel, D. E., Highly Water-Stable Polymer–Perovskite Nanocomposites. *ACS Appl. Mater. Interfaces* **2021,** *13* (49), 59252-59262.
7. Jiang, G.; Guhrenz, C.; Kirch, A.; Sonntag, L.; Bauer, C.; Fan, X.; Wang, J.; Reineke, S.; Gaponik, N.; Eychmüller, A., Highly Luminescent and Water-Resistant CsPbBr3–CsPb2Br5 Perovskite Nanocrystals Coordinated with Partially Hydrolyzed Poly(methyl methacrylate) and Polyethylenimine. *ACS Nano* **2019,** *13* (9), 10386-10396.
8. Hsieh, Y.-T.; Lin, Y.-F.; Liu, W.-R., Enhancing the Water Resistance and Stability of CsPbBr3 Perovskite Quantum Dots for Light-Emitting-Diode Applications through Encapsulation in Waterproof Polymethylsilsesquioxane Aerogels. *ACS Appl. Mater. Interfaces* **2020,** *12* (52), 58049-58059.
9. Ravi, V. K.; Saikia, S.; Yadav, S.; Nawale, V. V.; Nag, A., CsPbBr3/ZnS Core/Shell Type Nanocrystals for Enhancing Luminescence Lifetime and Water Stability. *ACS Energy Lett.* **2020,** *5* (6), 1794-1796.
10. Li, S.; Lei, D.; Ren, W.; Guo, X.; Wu, S.; Zhu, Y.; Rogach, A. L.; Chhowalla, M.; Jen, A. K. Y., Water-resistant perovskite nanodots enable robust two-photon lasing in aqueous environment. *Nat. Commun.* **2020,** *11* (1), 1192.
11. Chen, K.; Qi, K.; Zhou, T.; Yang, T.; Zhang, Y.; Guo, Z.; Lim, C.-K.; Zhang, J.; Žutic, I.; Zhang, H.; Prasad, P. N., Water-Dispersible CsPbBr3 Perovskite Nanocrystals with Ultra-Stability and its Application in Electrochemical CO2 Reduction. *Nanomicro Lett.* **2021,** *13* (1), 172.
12. Li, Z.; Hu, Q.; Tan, Z.; Yang, Y.; Leng, M.; Liu, X.; Ge, C.; Niu, G.; Tang, J., Aqueous Synthesis of Lead Halide Perovskite Nanocrystals with High Water Stability and Bright Photoluminescence. *ACS Appl. Mater. Interfaces* **2018,** *10* (50), 43915-43922.

13. Lee, S. M.; Jung, H.; Park, W. I.; Lee, Y.; Koo, E.; Bang, J., Preparation of Water-Soluble CsPbBr3 Perovskite Quantum Dot Nanocomposites via Encapsulation into Amphiphilic Copolymers. *ChemistrySelect* **2018,** *3* (40), 11320-11325.
